# Prognostic and predictive value of interstitial lung abnormalities and EGFR mutation status in patients with non-small cell lung cancer

**DOI:** 10.1186/s40644-024-00712-9

**Published:** 2024-05-23

**Authors:** Xiaoli Xu, Min Zhu, Zixing Wang, Jialu Li, Tao Ouyang, Cen Chen, Kewu Huang, Yuhui Zhang, Yanli L. Gao

**Affiliations:** 1 Department of Radiology, Beijing Chao-Yang Hospital, Capital Medical University, No. 8 Gongtinan Road, Beijing, 100020 China; 2 Department of Pulmonary and Critical Care Medicine, Beijing Institute of Respiratory Medicine, Beijing Chao-Yang Hospital, Capital Medical University, No. 8 Gongtinan Road, Beijing, 100020 China; 3 Institute of Basic Medical Sciences, School of Basic Medicine, Chinese Academy of Medical Sciences, Peking Union Medical College, Beijing, China; 4 Department of Radiology, Beijing Nuclear Industry Hospital, Beijing, China

**Keywords:** X-ray computed tomography, Lung cancer, EGFR, Interstitial lung abnormality, Overall survival

## Abstract

**Background:**

To determine the predictive value of interstitial lung abnormalities (ILA) for epidermal growth factor receptor (EGFR) mutation status and assess the prognostic significance of EGFR and ILA in patients with non-small cell lung cancer (NSCLC).

**Methods:**

We reviewed 797 consecutive patients with a histologically proven diagnosis of primary NSCLC from January 2013 to October 2018. Of these, 109 patients with NSCLC were found to have concomitant ILA. Multivariate logistic regression analysis was used to identify the significant clinical and computed tomography (CT) findings in predicting EGFR mutations. Cox proportional hazard models were used to identify significant prognostic factors.

**Results:**

EGFR mutations were identified in 22 of 109 tumors (20.2%). Multivariate analysis showed that the models incorporating clinical, tumor CT and ILA CT features yielded areas under the receiver operating characteristic curve (AUC) values of 0.749, 0.838, and 0.849, respectively. When combining the three models, the independent predictive factors for EGFR mutations were non-fibrotic ILA, female sex, and small tumor size, with an AUC value of 0.920 (95% confidence interval[CI]: 0.861–0.978, *p* < 0.001). In the multivariate Cox model, EGFR mutations (hazard ratio = 0.169, 95% CI = 0.042–0.675, *p* = 0.012; 692 days vs. 301 days) were independently associated with extended overall survival compared to the wild-type.

**Conclusion:**

Non-fibrotic ILA independently predicts the presence of EGFR mutations, and the presence of EGFR mutations rather than non-fibrotic ILA serves as an independent good prognostic factor for patients with NSCLC.

## Introduction

Interstitial lung abnormalities (ILAs) are increasingly recognized and identified in populations screened for lung cancer, with studies reporting their presence in 4-10% of cigarette smokers undergoing lung cancer screening [1–3], making them an important comorbidity in patients with lung cancer. According to the Fleischner Society, ILAs are radiological abnormalities incidentally detected on chest computed tomography (CT) in patients without a history of interstitial lung disease (ILD) with the potential for progression to idiopathic pulmonary fibrosis (IPF) or other fibrotic ILDs [1, 4]. Multiple large cohort studies have demonstrated a correlation between ILAs and an elevated incidence of lung cancer, heightened risk of all-cause mortality, and increased pulmonary complications associated with cancer treatment in patients [2, 5, 6]. Therefore, it is important to fully elucidate the relationship between ILAs and lung cancer before administering treatment in clinical practice.

With an increasing understanding of the molecular mechanisms underlying lung cancer, the treatment approach for non-small cell lung cancer (NSCLC) has evolved to prioritize the identification of specific oncogenic driver mutation subtypes, especially epidermal growth factor receptor (EGFR) mutations, which are the most common gene mutations and indicate longer overall survival for patients with NSCLC [7, 8]. Detection of EGFR mutation status typically requires invasive sampling of tumor tissue by surgery or biopsy. Inspired by genomics and tumor heterogeneity, thorough data mining of CT images has the potential to improve clinical decision-making [9]. Studies have demonstrated that the CT features of tumors could help identify the EGFR mutation status in patients [9–11]. A low prevalence of EGFR mutations has been previously reported in patients with lung cancer with usual interstitial pneumonia (UIP) [12]. However, the correlation between CT features of ILAs and EGFR mutations in NSCLC with coexisting ILAs has not yet been fully elucidated.

Studies have reported that patients with lung cancer and ILAs exhibit a lower 5-year overall survival (OS) rate than patients without ILA, yet demonstrate a higher 5-year OS rate than patients with IPF, and both fibrotic and non-fibrotic components of ILAs are associated with poor OS in patients with operable lung cancer [13, 14]. Nevertheless, there is a scarcity of studies that have concurrently examined EGFR mutations and ILAs in the assessment of prognosis among patients with lung cancer, particularly those in advanced stages. Hence, the objective of this study was to conduct a retrospective analysis of the predictive capability of ILA features on CT images for EGFR mutations, and to assess the prognostic significance of ILA and EGFR mutation status in patients with NSCLC with concurrent ILAs.

## Methods

### Study population

**Fig. 1:**
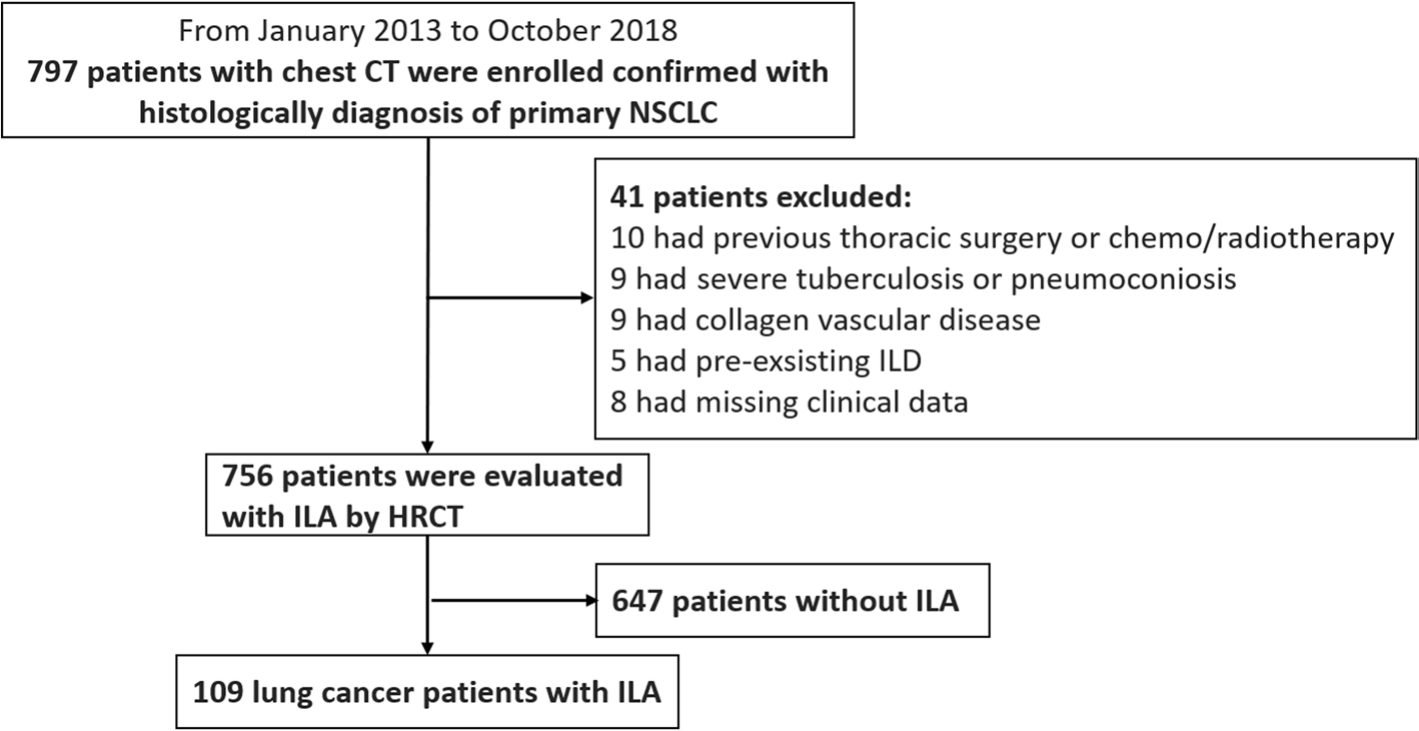
Flowchart of the selection of the study population and the exclusion criteria. CT, computed tomography; HRCT, high-resolution CT; ILA, interstitial lung abnormality; ILD, interstitial lung disease; NSCLC, non-small cell lung cancer

The Ethics Committees of Beijing Chao-Yang Hospital, Capital Medical University (no. 2021-ke-443) approved this retrospective, single-center study and waived the requirement for informed consent. A total of 797 consecutive patients with a histologically proven diagnosis of primary NSCLC underwent chest high-resolution CT (HRCT) between January 2013 and October 2018 at our hospital. The exclusion criteria were as follows: (i) previous thoracic surgery, radiation therapy, or chemotherapy; (ii) severe tuberculosis or pneumoconiosis; (iii) history of collagen vascular disease; (iv) preexisting ILD; and (v) missing clinical data. The HRCT images of these patients were reviewed retrospectively by two pulmonary radiologists (X-L X and Y-L G, with 10 and 25 years of experience, respectively) to identify the radiological evidence of concomitant ILA. According to the criteria of the Fleischner Society, ILAs are diagnosed as non-dependent ground-glass opacities (GGO), reticular abnormalities, architectural distortion, non-emphysematous cysts, honeycombing, and traction bronchiectasis that affect > 5% of any lung zone [4, 15]. Finally, 109 patients with concomitant lung cancer and ILA were included in this study (Fig. 1). Histological diagnosis was based on the World Health Organization classification published in 2021 [16] and TNM staging of lung cancer was according to the eighth edition [17], respectively. Molecular analysis of the EGFR mutation status was performed using polymerase chain reaction-based amplification-refractory mutation system analysis with the Human EGFR Gene Mutations Detection Kit (Beijing ACCB Biotech, Beijing, China). Smoking status was determined by reviewing medical records and was quantified by pack-years.

### CT evaluation by radiologists

CT images were obtained for all patients in the supine position at the end of inspiration using a variety of CT units. Images were reconstructed with contiguous 1–2 mm sections using a high-resolution reconstruction algorithm for analysis. Two chest radiologists independently evaluated the CT images in random order without any clinical or pathologic information. Image analysis was performed using the indicated window settings (lung: width, 1500 HU; level, -400 HU; mediastinum: width, 400 HU; level, 40 HU) for axial images on a picture archiving and communication system; radiologists were allowed to moderately change the default window settings for ease of assessment.

ILAs were subcategorized as non-subpleural, subpleural nonfibrotic, and subpleural fibrotic ILA [4], with subpleural fibrotic ILA corresponding to a probable usual interstitial pneumonia pattern [18]; non-subpleural ILA and subpleural nonfibrotic ILA were defined as non-fibrotic ILA in this study. The distribution, overall extent of the lesions, and extent of each ILA lesion were further evaluated, and disagreements concerning the scores were resolved by consensus. The predominant distribution included peripheral, peribronchovascular, and mixed patterns. The extent of CT findings of ILA in each case was scored using a four-point scale (score of 1, 5–25% involvement; score of 2, 26–50% involvement; score of 3, 51–75% involvement; and score of 4, 76–100% involvement) [1]. The extent of the ILA findings was further assessed in six lung zones (upper, middle, and lower zones of both lungs) on CT images. The division of the upper, middle, and lower lung zones was determined based on the levels of the inferior aortic arch and right inferior pulmonary vein [4]. Readers scored the lung fields that showed abnormalities in each of the six zones based on GGO, reticulation, and honeycombing. The average of the six lung zones was used to calculate the percentage of the whole lungs. Traction bronchiectasis was assessed by summing the number of bronchiectasis-affected pulmonary segments [19].

Morphological CT features of lung tumors were also analyzed, including location, size (maximum long-axis diameter), shape (round, somewhat irregular, irregular), density (solid, part-solid, GGO), margin (lobulation, spiculation), internal (vacuole sign, lumen, cavity, air bronchogram, bronchial cut-off sign), surrounding structures (vascular convergence, pleural traction, halo sign), and associated findings (pleural fluid). Previously published evaluation methods were used in this analysis [10, 20]. Disagreements regarding the CT features of lung cancer were resolved by consensus.

### Statistical analysis

All statistical analyses were performed using SPSS version 20.0. Data are presented as means, medians, counts, and percentages, where appropriate. Clinical and CT feature variables between the EGFR mutation and wild-type groups were compared using X^2^ or Fisher’s test, Student’s t-test, or the Mann–Whitney U test, where appropriate. Multiple logistic regression analysis was used to assess the value of clinical features, tumor CT findings, and ILA CT features in predicting EGFR mutations. Significant factors in the univariate analysis were identified as potential covariates in a multivariate logistic regression model using the forward likelihood ratio test. A receiver operating characteristic (ROC) curve was used to estimate the significant predictors of EGFR identification. The area under the curve (AUC) was calculated to determine the predictive capability. A *p* value of less than 0.05 was considered to indicate a significant difference.

The probabilities of OS at three and five years after diagnosis were estimated using the Kaplan–Meier method. Multivariate analysis was performed using the Cox proportional hazards model to evaluate the hazard ratios (HR) for OS probabilities with 95% confidence intervals (CI).

## Results

### Correlation of EGFR mutation status with clinical characteristics

The clinical features of the patients are shown in Table 1. EGFR mutations were identified in 22 of the 109 tumors (20.2%). EGFR mutations were more frequently observed in women (63.64%) than in men (36.36%) (*p* < 0.001), and in non-smokers (68.18%) than in smokers (31.82%) (*p* < 0.001). No difference in the average age was observed between the EGFR mutant and wild-type groups (*p* = 0.421). Most EGFR mutations (95.45%) were found adenocarcinomas (*p* = 0.007). Lung cancers with EGFR mutations mostly presented with an early TNM stage (*p* = 0.034).

**Table 1:** Association between Clinical features and EGFR mutation status

Characteristic	Total	EGFR (+)	EGFR (-)	*P* value
**No. of patients**	109	22 (20.18)	87 (79.82)	
**Sex**				
Male	83 (76.15)	8 (36.36)	75 (86.21)	< 0.001
Female	26 (23.85)	14 (63.64)	12 (13.79)	
**Age (y)**^a^	66.07 ± 8.14	64.82 ± 7.04	66.39 ± 8.41	0.421
**Smoking history**				< 0.001
Never a smoker	37 (33.94)	15 (68.18)	22 (25.29)	
Smoker	72 (66.06)	7 (31.82)	65 (74.71)	
**Smoking amount (pack-year)**^b^	25(0,45)	0(0,25)	30(0,45)	0.007
**Histologic finding**				0.007
Adenocarcinoma	78 (71.56)	21 (95.45)	57 (65.52)	
Squamous cell carcinoma	27 (24.77)	1 (4.55)	26 (29.88)	
Others	4 (3.67)	0 (0)	4 (4.60)	
**TNM staging**				0.034
I + II	21(19.27)	8 (36.36)	13 (14.94)	
III + IV	88 (80.73)	14 (63.64)	74 (85.06)	
**Diagnostic technique**				
Surgery	24 (22.02)	11 (50)	13 (14.94)	
CT-guided biopsy	82 (75.23)	11 (50)	71 (81.61)	
others	3 (2.75)	0 (0)	3 (3.45)	

Unless otherwise indicated, data are numbers of patients, with the percentages in parentheses

*EGFR* Epidermal growth factor receptor

^a^Data are means ± standard deviations

^b^Data are the median with interquartile range in parentheses

### Correlation of EGFR mutation status with CT features of ILA and tumors

**Fig. 2:**
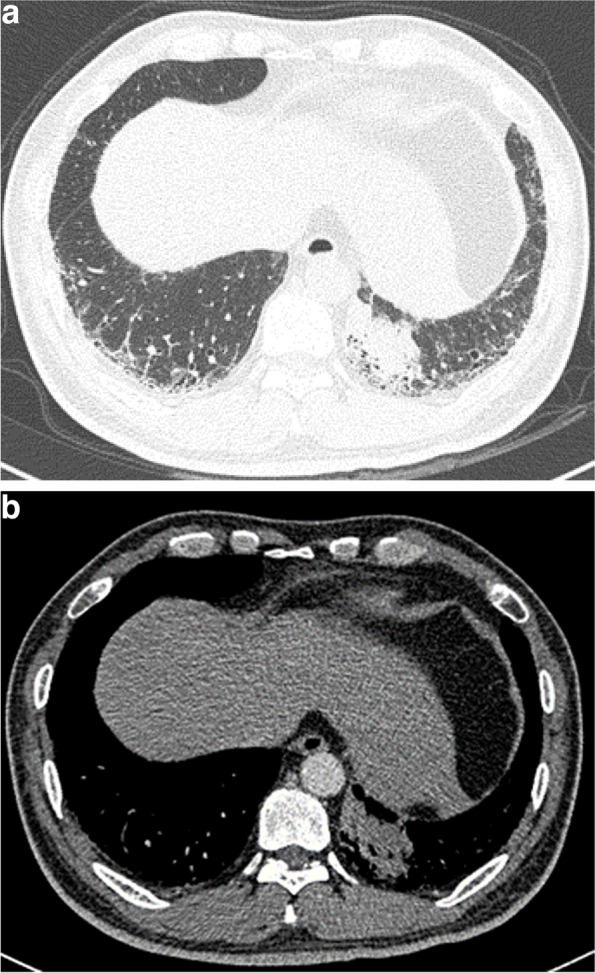
A 54-year-old male smoker with stage IV adenocarcinoma located in the left lower lobe. Pre-treatment chest CT (**a**) demonstrated ground-glass opacity, reticulation, traction bronchiectasis, and honeycombing predominantly located in bilateral lower lobes, in accordance with subpleural fibrotic ILA. The tumor was located in ILAs and presented the lobulation sign (**a**, **b**). The patient underwent fine needle aspiration biopsy, with a pathological diagnosis of adenocarcinoma without EGFR mutation. He was died 300 days after the diagnosis of lung cancer. CT, computed tomography

**Fig. 3:**
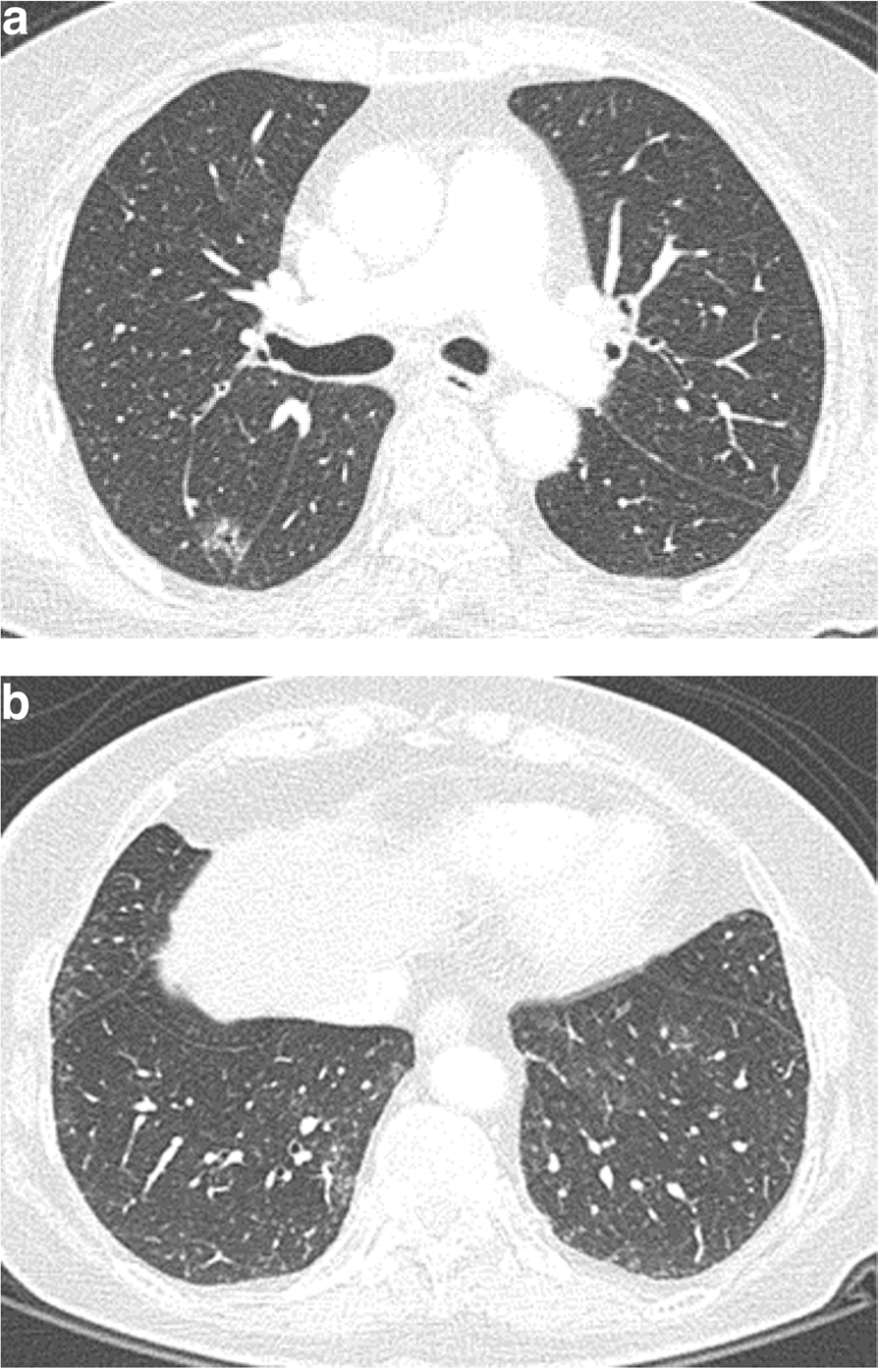
A 69-year-old female nonsmoker with stage I adenocarcinoma located in the right upper lobe. Pre-treatment chest CT demonstrated the tumor with signs of lobulation, spiculation, vacuole and pleural traction (**a**). Ground-glass opacity and reticulation were observed in bilateral lower lobes without subpleural predominance, in accordance with non-fibrotic ILA (**b**). The patient underwent surgery, and a pathological diagnosis of adenocarcinoma with EGFR mutation was made. She was still alive at the end of follow-up. CT, computed tomography

The CT features of the ILA included in the univariate analysis for prediction of EGFR mutations are presented in Table 2. EGFR mutation rates were significantly lower in subpleural fibrotic ILA than in non-fibrotic ILA (*p* < 0.001) (Figs. 2 and 3). The ILAs in the EGFR mutant group were less distributed in the subpleural zone. The scores for reticulation in every zone and honeycombing in the middle and lower zones of the EGFR mutant group were significantly lower than those of the wild-type group. The degree of traction bronchiectasis was also less in the EGFR mutant group (*p* < 0.001). No significant difference was noted in the GGO score of any lung zone between the EGFR mutant and wild-type groups.

**Table 2:** CT Findings of ILA and Their Association with EGFR mutation

**Estimation of extent **	**EGFR (+)**	**EGFR (-)**	*P***value**
**Subcategories**	22	87	<0.001
Subpleural fibrotic ILA	2 (9.10)	55 (63.22)	
Subpleural nonfibrotic ILA	10 (45.45)	18 (20.69)	
Non-subpleural ILA	10 (45.45)	14 (16.09)	
**Total score**^a^	1.23±0.53	1.28±0.48	0.580
**Distribution**			0.012
Subpleural	17 (77.27)	79 (90.80)	
Peribronchovascular	5 (22.73)	3 (3.45)	
Mixed	0	5 (5.75)	
**Score of ILA findings**^a^			
Upper GGO	4.11±9.90	4.01±6.80	0.154
Middle GGO	7.61±10.41	10.13±10.98	0.068
Lower GGO	14.89±14.06	17.23±15.41	0.433
Upper reticulation	2.61±6.70	7.99±9.19	<0.001
Middle reticulation	3.75±4.41	12.66±11.67	<0.001
Lower reticulation	8.11±6.45	19.53±15.32	<0.001
Upper honeycombing	0.00±0.00	1.28±3.63	0.068
Middle honeycombing	0.11±0.53	2.83±5.67	0.001
Lower honeycombing	0.41±1.46	7.50±12.56	<0.001
Tractive bronchiectasis	1.91±3.90	7.15±5.46	<0.001

Unless otherwise indicated, data are numbers of patients, with the percentages in parentheses

*ILA* Interstitial lung abnormality, *EGFR* Epidermal growth factor receptor

^a^Data are means ± standard deviations

Regarding the CT features of the lung tumors (Table 3), the tumor diameter of the EGFR mutation group was significantly smaller than that of EGFR wild-type group (*p* < 0.001). Spiculation (*p* = 0.043) and pleural traction (*p* = 0.001) were more frequently observed in the EGFR mutant group than in the wild type group, whereas pleural fluid was less frequently observed in the EGFR mutant group. In addition, tumors located in ILA had a lower rate of EGFR mutations (*p* < 0.001). Other CT features, such as location, shape, density, vacuole sign, lumen, cavity, air bronchogram, bronchial cutoff sign, lobulation, vascular convergence, and halo sign, showed no significant differences between the two groups.

**Table 3:** CT Findings of tumor and their association with EGFR

Estimation of tumor	EGFR (+)	EGFR (-)	*P* value
**Diameter(cm)**^a^	3.10 ± 1.15	5.26 ± 2.62	< 0.001
**Spiculation (+)**	19(86.36)	55(63.22)	0.043
**Pleural traction (+)**	19(86.36)	41(47.13)	0.001
**Pleural fluid (+)**	6(27.27)	48(55.17)	0.030
**Tumor located in ILA**	4(18.18)	54(62.07)	< 0.001

Unless otherwise indicated, data are numbers of patients, with the percentages in parentheses

*EGFR* Epidermal growth factor receptor, *ILA* Interstitial lung abnormality

^a^Data are means ± standard deviations

### Multivariable analysis and receiver operating characteristic curve analysis

**Fig. 4:**
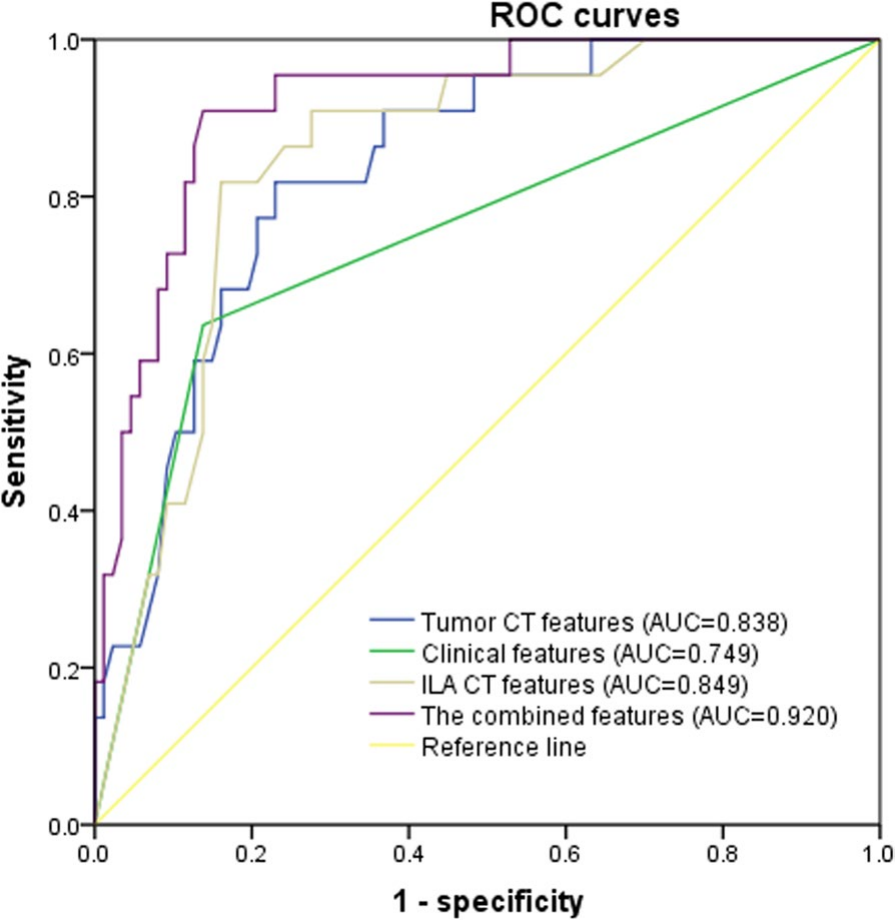
Receiver operating characteristic curves applied to predict EGFR mutation status. The curves are shown for models based on clinical features, tumor CT features, ILA CT features and integrated features. AUC, area under the ROC curve; CT, computed tomography; EGFR, epidermal growth factor receptor; ILA, interstitial lung abnormality; ROC, receiver operating characteristic

In the multivariate analysis, for the model with clinical features, sex (OR = 0.091, 95% CI: 0.032–0.264; *p* < 0.001) was an independent predictive factor for the presence of EGFR mutations after adjusting for smoking history, histologic findings, and TNM staging, with an AUC value of 0.749 (95% CI: 0.622–0.877, *p* < 0.001). The model with tumor CT features showed that tumor located in ILA (OR = 0.156, 95% CI: 0.046–0.529; *p* = 0.003) and tumor diameter (OR = 0.592, 95% CI: 0.415–0.846; *p* = 0.004) were independent predictors of EGFR mutation; the corresponding AUC value was 0.838 (95% CI: 0.754–0.922, *p* < 0.001). The model with ILA CT features indicated that subpleural fibrotic ILA (OR = 0.097, 95% CI: 0.019–0.483; *p* = 0.004) and middle reticulation (OR = 0.890, 95% CI: 0.803–0.987; *p* = 0.027) were independent predictors of EGFR mutations, with an AUC value of 0.849 (95% CI: 0.768–0.930, *p* < 0.001). When combining the three models, the significantly independent predictors for EGFR mutation were subpleural fibrotic ILA (OR = 0.169, 95% CI: 0.030–0.964; *p* = 0.043), sex (OR = 0.141, 95% CI: 0.035–0.557; *p* = 0.005) and tumor diameter (OR = 0.637, 95% CI: 0.429–0.944; *p* = 0.025), giving a predictive value for EGFR mutations with the AUC value of 0.920 (95% CI: 0.861–0.978, *p* < 0.001), a diagnostic sensitivity of 90.9% and a specificity of 86.2% (Fig. 4).

**Fig. 5:**
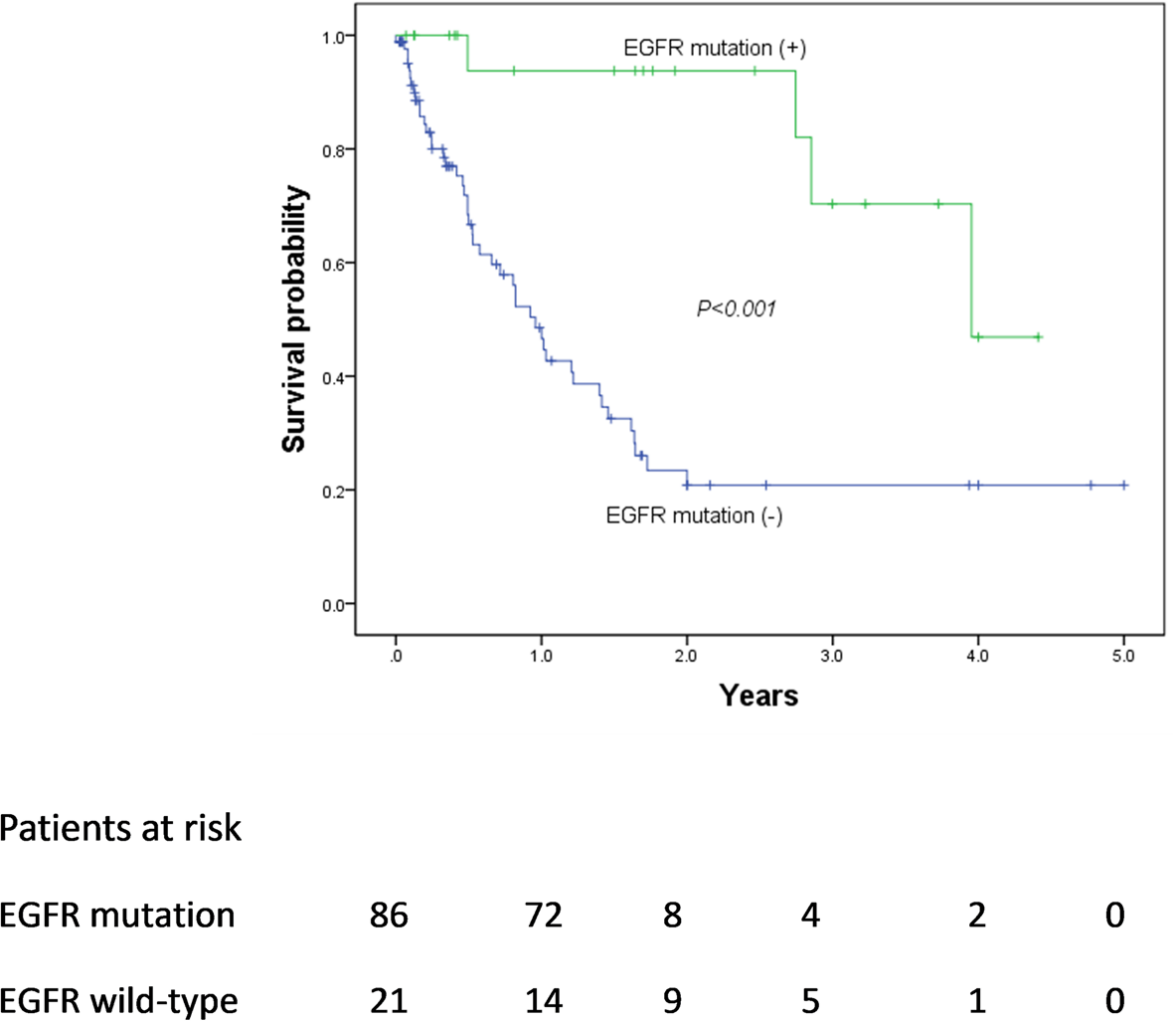
Overall survival according to the EGFR mutation status. The log-rank test showed that patients with EGFR mutations had a good overall survival compared to patients without EGFR mutations (*p* < 0.001). EGFR, epidermal growth factor receptor

The Kaplan–Meier curves for OS based on EGFR status are shown in Fig. 5. Patients with EGFR mutations had a longer OS than those without EGFR mutations (*p* < 0.001). In the univariate analysis of OS after diagnosis, sex, smoking status, subpleural fibrotic ILA, EGFR status, TNM staging, histologic findings, pleural traction, and presence of pleural fluid were predictive factors. In the multivariate analysis, EGFR mutation (HR = 0.169, 95% CI = 0.042–0.675, *p* = 0.012; 692 days vs. 301 days), TNM stage III–IV (HR = 14.819, 95% CI = 1.936–113.407, *p* = 0.009; 336 days vs. 580 days), and presence of pleural fluid (HR = 2.796, 95% CI = 1.347–5.803, *p* = 0.006; 274 days vs. 489 days) were independent prognostic factors for OS (Table 4).

**Table 4:** Univariate and multivariate Cox analysis for overall survival

Variables	Univariate analysis for OS		Multivariate analysis for OS	
	HR (95% CI)	*P* value	HR (95% CI)	*P* value
**Sex** (male vs. female)	3.066 (1.366–6.884)	0.007	1.091 (0.367–3.243)	0.875
**Smoking status** (current or former smoker vs. never)	2.121 (1.101–4.087)	0.025	1.384 (0.595–3.220)	0.450
**ILA subcategory** (subpleural fibrotic ILA vs. non-fibrotic ILA)	2.065 (1.156–3.690)	0.014	1.282 (0.678–2.424)	0.444
**EGFR status** (mutated vs. wild)	0.165 (0.059–0.466)	0.001	**0.169 (0.042–0.675)**	**0.012**
**TNM staging** (III-IV vs. I-II)	16.414 (2.258-119.292)	0.006	**14.819 (1.936-113.407)**	**0.009**
**Histologic findings** (adenocarcinoma vs. non-adenocarcinoma)	0.301 (0.156–0.581)	<0.001	0.612 (0.293–1.281)	0.193
**Pleural traction (+)**	0.503 (0.285–0.889)	0.018	0.802 (0.428–1.503)	0.491
**Pleural fluid (+)**	3.740 (2.005–6.977)	<0.001	**2.796 (1.347–5.803)**	**0.006**

*OS* Overall survival, *HR* Hazard ratio, *CI* Confidence interval, *EGFR* Epidermal growth factor receptor, *ILA* Interstitial lung abnormality

## Discussion

The evaluation of EGFR mutations and associated risk factors of OS in patients with NSCLC is crucial for clinicians to determine the appropriate course of treatment, monitor treatment efficacy, and predict patient outcomes. Previous investigations have revealed an unfavorable prognosis for ILA in patients with lung cancer [13, 14, 21, 22]. Our previous study comprehensively analyzed the prevalence of ILAs, clinical characteristics, and prognosis of patients with newly diagnosed NSCLC [22]. Based on this foundation, in this study we have expanded the investigation into the subgrouping of ILAs through CT analysis, exploring their potential predictive value for determining the EGFR status of tumors and long-term prognosis. This showed that the CT features of ILAs were predictive of the EGFR mutation status, and the combined clinical and imaging features could further improve the predictive ability for EGFR mutation status. The OS was shorter when patients with lung cancer presented with TNM stage III–IV, EGFR wild-type mutation, and pleural fluid. Moreover, non-fibrotic ILA was identified as an independent predictor of EGFR but not as independent prognostic factor for OS.

EGFR mutations have been observed at different rates in American and Asian populations, ranging from 28% in American non-smokers to 68% in Asian non-smokers [23]. This study found that the EGFR mutation rate was 20.2% in a Chinese population with lung cancer and coexistent ILA, and the mutation frequency in patients with subpleural fibrotic ILA (3.5%) was lower than that in those with non-fibrotic ILA (38.5%), which is similar to findings in earlier studies in the Japanese population [12, 24]. Although some researchers have reported a high risk of acute exacerbation caused by molecular-targeted agents among patients with lung cancer and coexisting ILD [25, 26], tyrosine kinase inhibitors are still administered to selected patients, especially patients with NSCLC and ILA [26, 27].

CT identification of the EGFR mutation status has the potential to inform physicians when tailoring treatment strategies for patients [9, 10]. The clinical characteristics and CT features of tumors with concurrent ILAs in this study aligned with the findings reported in previous studies that did not consider ILAs [9, 11]. EGFR mutations were more frequently observed in women, non-smokers, early TNM stage, and were associated with a higher frequency of adenocarcinoma. CT features of the EGFR mutation group, compared to the wild-type group, were small tumors, with spiculation, pleural traction, and less pleural fluid. Additionally, fewer tumors were located in the ILA for the EGFR mutant group than in the EGFR wild-type group. The distribution and extent of ILA findings were analyzed and quantified in detail in this study，this has been discussed less frequently in prior studies. Our results showed that the ILAs of the EGFR mutant group were less distributed in the subpleural zone, and the reticulation score of every zone and the honeycombing score of the middle and lower zones were all significantly lower in the EGFR mutant group than in the wild-type group. Traction bronchiectasis was also less frequently observed in the EGFR mutant group. Multivariable regression analyses demonstrated that the combination of clinical variables, tumor CT features, and ILA CT features significantly improved the predictive ability, with non-fibrotic ILA and small tumors as significant independent predictive factors for EGFR mutation. EGFR mutations occurred less frequently in subpleural fibrotic ILA, a finding that corresponds to previous studies that reported a low prevalence of EGFR mutations in patients with lung cancer and UIP or IPF [12, 24]. Watanabe et al. [28] reported that the origin of lung cancer in the subpleural region was correlated with UIP. Sakuma et al. [29] further demonstrated that alveolar epithelial cells that have undergone epithelial-to-mesenchymal transition (EMT) may contribute to the formation of fibroblastic foci in IPF, whereas EGFR-mutant lung adenocarcinoma cells that have undergone EMT lack EGFR dependency. This may elucidate the potential pathophysiological mechanisms underlying subpleural fibrotic ILA, which is mostly accompanied by wild-type EGFR lung cancer.

Previous studies have demonstrated that the detection of ILA in patients with early or advanced NSCLC is correlated with a decreased OS rate [6, 14, 22]. In addition, subpleural fibrotic ILAs are linked to a lower survival rate than non-fibrotic ILAs [14, 15, 30]; however, EGFR mutation status was not included in these analyses. Researchers have reported EGFR mutations as a good prognostic factor for advanced lung cancer, but not for operable early stage lung adenocarcinoma [8, 11]. In our study, 80.7% of patients were diagnosed with stage III-IV lung cancer, and EGFR mutation status was incorporated into the multivariate analysis and identified as an independent prognostic factor for patients with lung cancer and ILA, after excluding those with subpleural fibrotic ILA. Similarly, Kanaji et al. [24] reported that the lack of EGFR mutations and the presence of IPF were unfavorable prognostic factors for progression-free survival and OS. Given the significantly better prognosis of patients with ILAs compared to those with IPF [4, 15, 24], this may explain why subpleural fibrotic ILA is not an independent prognostic factor. Overall, acknowledging the prognostic implications of EGFR mutations in lung cancer with ILAs can greatly influence clinical decision-making.

This study had several limitations. First, the retrospective nature of this single-center observational study resulted in a natural selection bias and missing data. A prospective study with multicenter data is necessary in the future. Second, due to the low prevalence of EGFR mutations in patients with ILAs, the sample size was relatively small in this study for the EGFR mutation group; however, the association between EGFR mutations and CT features of ILAs is of significant clinical and statistical importance. Third, 78% (85/109) of the patients in the study underwent biopsy procedures to obtain samples, as most patients presented with stage III-IV lung cancer. However, both the adequacy of the sample and accuracy of the diagnosis of EGFR mutations were rigorously confirmed. Finally, the assessment primarily concentrated on the baseline ILA, without further dynamic observation of the ILA or examination of its connection to OS. Future research should involve additional investigations in this area.

## Conclusions

The presence of non-fibrotic ILA is associated with an increased incidence of EGFR mutations, whereas the presence of EGFR mutations rather than ILA is an independent positive prognostic factor for patients with NSCLC. Consequently, understanding the predictive significance of ILA for EGFR mutations and the prognostic value of EGFR status can assist clinicians and radiologists in improving their understanding and evaluation of the long-term prognosis of patients with NSCLC and ILAs.

## Data Availability

The datasets used and analyzed during the study are available from the corresponding authors on reasonable request.

## Abbreviations

AUC: Area under the receiver operating characteristic curve
CI: Confidence interval
CT: Computed tomography
EGFR: Epidermal growth factor receptor
GGO: Ground-glass opacity
HR: Hazard ratio
HRCT: High-resolution computed tomography
ILA: Interstitial lung abnormality
ILD: Interstitial lung disease
IPF: Idiopathic pulmonary fibrosis
NSCLC: Non-small cell lung cancer
OS: Overall survival
ROC: Receiver operating characteristic
UIP: Usual interstitial pneumonia
